# Flyway‐scale analysis reveals that the timing of migration in wading birds is becoming later

**DOI:** 10.1002/ece3.8130

**Published:** 2021-09-28

**Authors:** Thomas O. Mondain‐Monval, Matt Amos, Jamie‐Leigh Chapman, Andrew MacColl, Stuart P. Sharp

**Affiliations:** ^1^ Lancaster Environment Centre Lancaster University Lancaster UK; ^2^ Wrightington, Wigan and Leigh NHS Foundation Trust Wigan UK; ^3^ Life Science, University Park Nottingham UK

**Keywords:** birds, climate change, continental scale, eBird, migration, phenology, waders, weather

## Abstract

1. Understanding the implications of climate change for migratory animals is paramount for establishing how best to conserve them. A large body of evidence suggests that birds are migrating earlier in response to rising temperatures, but many studies focus on single populations of model species.
2. Migratory patterns at large spatial scales may differ from those occurring in single populations, for example because of individuals dispersing outside of study areas. Furthermore, understanding phenological trends across species is vital because we need a holistic understanding of how climate change affects wildlife, especially as rates of temperature change vary globally.
3. The life cycles of migratory wading birds cover vast latitudinal gradients, making them particularly susceptible to climate change and, therefore, ideal model organisms for understanding its effects. Here, we implement a novel application of changepoint detection analysis to investigate changes in the timing of migration in waders at a flyway scale using a thirteen‐year citizen science dataset (eBird) and determine the influence of changes in weather conditions on large‐scale migratory patterns.
4. In contrast to most previous research, our results suggest that migration is getting later in both spring and autumn. We show that rates of change were faster in spring than autumn in both the Afro‐Palearctic and Nearctic flyways, but that weather conditions in autumn, not in spring, predicted temporal changes in the corresponding season. Birds migrated earlier in autumn when temperatures increased rapidly, and later with increasing headwinds.
5. One possible explanation for our results is that migration is becoming later due to northward range shifts, which means that a higher proportion of birds travel greater distances and therefore take longer to reach their destinations. Our findings underline the importance of considering spatial scale when investigating changes in the phenology of migratory bird species.

## Graphical abstract

Millions of birds migrate annually providing valuable ecosystem services, but we know little about macroecological changes occurring due to climate change. Our results suggest that continental‐scale wading bird migration has become later over the last decade, contrary to many studies at the population‐level, suggesting that changes in the timing of bird migration might differ across spatial scales. More work is urgently needed to investigate large‐scale patterns in the phenology of migratory animals.

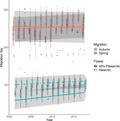

## 1 INTRODUCTION

The effects of anthropogenic climate change on migratory bird species have received much attention because there is a clear link to recent population declines, changes in phenology and distribution shifts (Gill et al., 2019; Root et al., 2003; Wilcove & Wikelski, 2008). Migrants travel in vast numbers between ecologically distinct geographic regions, thereby providing valuable ecosystem services (Viana et al., 2016; Wilcove & Wikelski, 2008). However, they are particularly susceptible to the effects of climate change because they are prone to different sources of pressure at each stage of their life cycle (Van Gils et al., 2016). Understanding the mechanisms driving migratory bird population trends is therefore extremely challenging.

One particularly well‐documented impact of climate change is a shift in migratory phenology (Gordo, 2007; Lehikoinen et al., 2004; Parmesan & Yohe, 2003). Most studies have shown that the timing of spring migration is becoming earlier, whereas in autumn, trends vary considerably between species (Adamík & Pietruszková, 2008; Gunnarsson & Tómasson, 2011; Lehikoinen et al., 2004; Parmesan & Yohe, 2003). However, many of these studies have focused on single populations of species, and this may result in larger‐scale patterns being missed (Kelly & Horton, 2016). For example, most studies are unable to account for individuals leaving study sites, and the assumption that patterns are similar in other populations is undermined by geographic differences in temperature change, migration routes, and timing (Chambers et al., 2014; Chmura et al., 2019; Gilroy et al., 2016). Furthermore, phenological responses can vary between species (Mayor et al., 2017; Newton, 2010) and across latitudinal gradients (Chmura et al., 2019). While population‐ and species‐level research is certainly valuable for identifying the drivers of phenological change or the key factors affecting endangered species, macroecological studies are vital to obtain a holistic understanding of the impacts of climate change (Horton et al., 2019; Kelly & Horton, 2016). This is important because the services that migratory species provide rely on the total number of birds migrating, and any changes in these numbers will have significant consequences for ecosystem functioning (Viana et al., 2016; Wilcove & Wikelski, 2008).

Early arrival to breeding regions is thought to be beneficial for breeding success by providing access to better territories, increasing the amount of time for reproduction, and improving chick recruitment (Kokko, 1999; Morrison et al., 2019). However, climate change has caused long‐term advances in the timing of peak insect abundance which, for many birds, have not been matched by the timing of breeding (Mayor et al., 2017). In some cases, these mismatches have led to reduced reproductive success and significant population declines (Both et al., 2006; Møller et al., 2008). In autumn, there is less consensus about the effects of climate change for the timing of migration than in spring. For example, while the timing of autumn migration has become later for short‐distance migrants, that of long‐distance migrants has advanced (Adamík & Pietruszková, 2008; Jenni & Kery, 2003; Newson et al., 2016). Differences in phenological change between spring and autumn migration are likely to have important implications for productivity and population trends (Halupka & Halupka, 2017).

Weather patterns have a major influence on the timing of migration. For example, individuals favor tailwinds when departing for migration after stationary periods (Shamoun‐Baranes, et al., 2010; Shamoun‐Baranes, Leyrer, et al., 2010) and have been shown to adjust their flight altitude to exploit the most favorable wind conditions (Senner et al., 2018). The impact of weather is likely to differ between spring and autumn migration because individuals are under less time pressure in autumn when there are no constraints associated with the timing of breeding (Conklin et al., 2013; McNamara et al., 1998; Møller et al., 2008). Therefore, birds may wait for favorable conditions (Conklin et al., 2013) or spend longer improving body condition prior to migration (Duijns et al., 2017). Moreover, the population‐level timing of migration is correlated with weather patterns in different regions throughout the life cycle (Gordo, 2007). This is likely to operate through the knock‐on influence of ground conditions on individual body condition (Duijns et al., 2017), the cues they provide to migrating birds regarding breeding site conditions (Forchhammer et al., 2002), and their effect on the duration of reproduction (Townsend et al., 2013). However, further research is needed to elucidate the effects of weather conditions on migration patterns at the flyway scale.

Migratory shorebirds or wading birds (hereafter “waders”) are a group in decline and of high conservation concern (AEWA, 2018). Many waders are migratory, they breed through a wide range of latitudes, and they are reliant on relatively specific and seasonal habitats (Haig et al., 2019; Piersma, 2007), all of which increase their susceptibility to climate change (Both et al., 2009). Furthermore, studies of wader phenology at breeding, wintering, and passage sites have shown contrasting trends with both advances and delays to the timing of migration (Adamík & Pietruszková, 2008; Meltofte et al., 2018; Murphy‐Klassen et al., 2005). Waders are therefore the ideal group in which to study the impact of climatic conditions on the timing of migration. Here, we use over ten years' worth of sightings from eBird, the Cornell Laboratory of Ornithology citizen science database (Sullivan et al., 2014), to investigate changes in the migratory phenology of waders in two major flyways. Specifically, we use a novel application of changepoint detection analysis to determine whether the phenology of migratory birds at a flyway scale has changed over time. Changepoint analysis is used to identify the point at which the statistical properties of a time series change, in this case changes in mean and variance (Killick & Eckley, 2014). We then investigate whether the timing of spring and autumn migration is correlated with changing weather conditions across global breeding and wintering distributions.

## 2 MATERIALS AND METHODS

### 2.1 eBird data

Sightings of all wader species classified by Birdlife as being migratory were downloaded from the eBird citizen science database (Sullivan et al., 2014). Analyses were restricted to the four major families Charadriidae, Haematopodidae, Recurvirostridae, and Scolopacidae, for which most data were available. The data were filtered to include only observations from 2003 to 2016. While eBird started in 2002, the database contains some historical observations which were not suitable for our analyses. Sightings were split into three major flyways based on longitude: the Nearctic Flyway (classified as 170°W to 24°W); the Afro‐Palearctic Flyway (as 26°W to 90°E); and the East Australian Flyway (as 91°E to 170°E; (Colwell, 2010)). These divisions were chosen following broadscale continental divides. However, the East Australian flyway had too few data and so we excluded it from subsequent analyses. Some species occurred in both the Nearctic and Afro‐Palearctic flyways; these populations were considered separately in the analyses because there are likely to be different selection pressures operating between flyways. We also removed species that do not carry out an intercontinental migration, such as some intra‐Africa migrants. Elsewhere in the methodology, “species” is used to mean “species by flyway.”

For each day in each year, we created a mean latitudinal location for each species by averaging the latitudes of all sightings reported. Observer bias may lead to species not being reported at latitudes in which they were present. To account for this, for each day, we determined (a) the number of times a species was seen at each latitude (latitudes were considered as one‐degree latitudinal bands) and (b) the total number of sightings of any wading bird species reported at any latitude. Therefore, for each day we had the number of sightings of a species in each latitudinal band and the total numbers of sightings of all species across all latitudinal bands. We then used the number of times a species was reported at each latitude as a proportion of the total number of sightings of any species seen across all latitudes to create a daily, weighted mean latitude for each species. For example, a species for which 100 sightings were reported from 35°N on a day in which 1,500 sightings of waders of all species were reported across all latitudes, was given a weighting of 100/1,500 for that latitude on that day. This proportion provided an index of the effort made to observe a species at a given latitude, relative to the total effort made to observe waders across all latitudes. Additionally, we removed any days on which the total number of sightings of all species within a flyway was less than five. This avoided biasing the data due to a relatively small number of observers being out on any given day (Johnston et al., 2019).

### 2.2 Changepoint analysis

We were interested in identifying changes in both spring and autumn migration. We define “spring migration” to be the movement of individuals northwards, toward the breeding grounds, with “autumn migration” referring to the movement south toward the nonbreeding grounds. In order to determine the timing of these migrations in each year, we identified significant shifts in the mean latitude of each species. We excluded the years of data that contained fewer than three hundred days of observations for each species within each flyway (in the Nearctic, 162 years in total from 38 different species were excluded; in the Afro‐Palearctic, 151 years from 26 different species), and considered each year individually. We then used changepoint detection analysis to detect these shifts.

Suppose that ${\left\{y_{t}\right\}}_{t=1,\ldots ,n}\sqrt{2}$ represents our daily mean latitudinal observations of a species over a one‐year period, where n is the number of observations for that year and *t* is the day of the year. Then, a changepoint in these data, “τ,” corresponds to a point in time such that the statistical properties of ${\left\{y_{t}\right\}}_{t=1,\ldots ,\tau }$, and ${\left\{y_{t}\right\}}_{t=\tau +1,\ldots ,n}$ differ in some way. A data set could contain multiple changepoints, which divide the data into segments; each of these segments will have some different statistical property. For example, if a data set contained changes in its mean, then each segment would have a different mean. There might be only one statistical property that changes, or there could be multiple properties. Figure S1 gives an example of three types of changepoint: (a) change in mean, (b) change in variance, and (c) change in both mean and variance. For an introduction to changepoint detection in an environmental setting, see Andersen et al. (2009).

**FIGURE 1:**
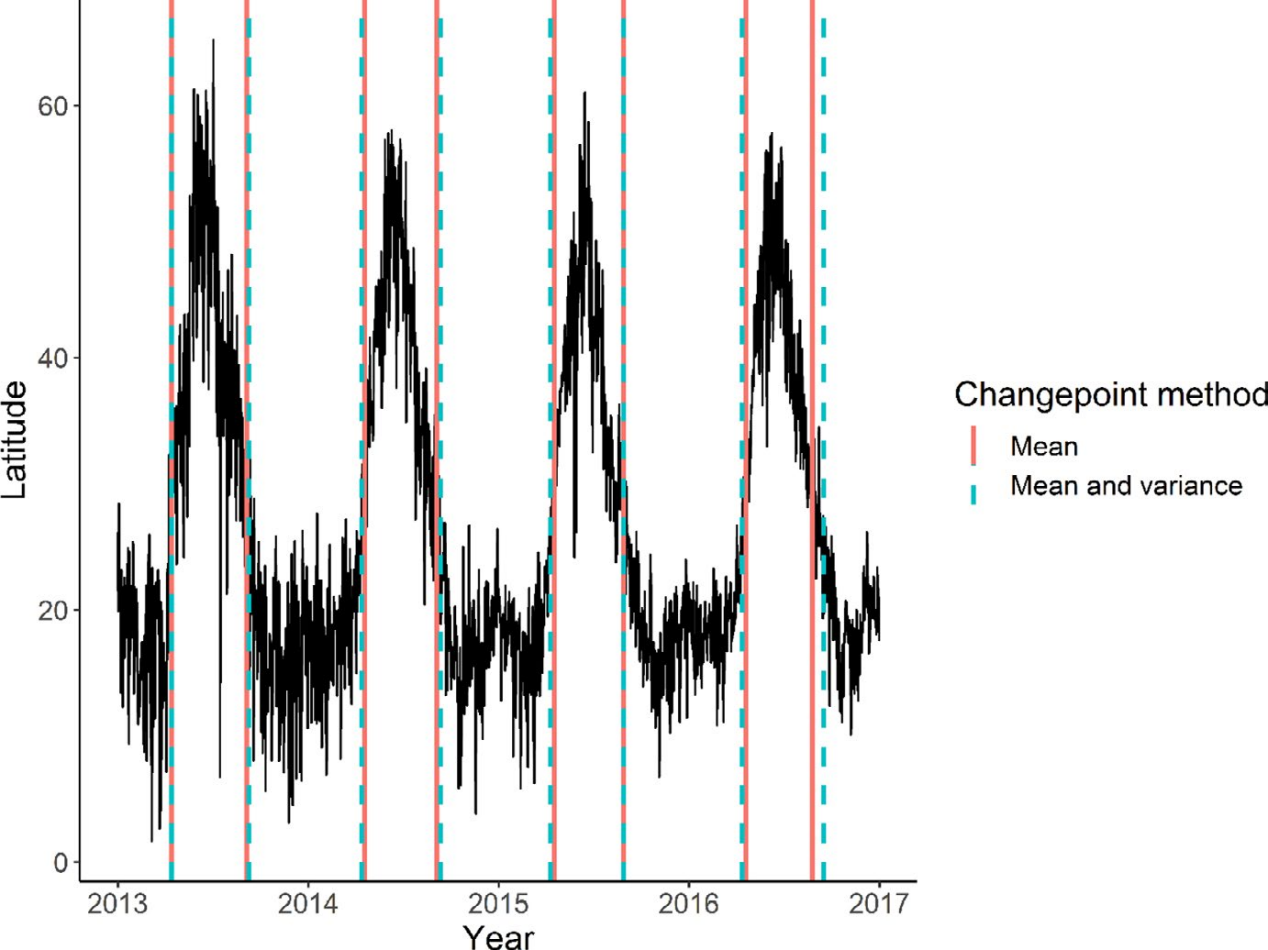
The mean daily latitude of common sandpipers *Actitis hypoleucos* in the Afro‐Palearctic flyway between 2013 and 2017 and a comparison of the migration days identified by the two changepoint detection methods, mean, and mean and variance combined

**FIGURE 2:**
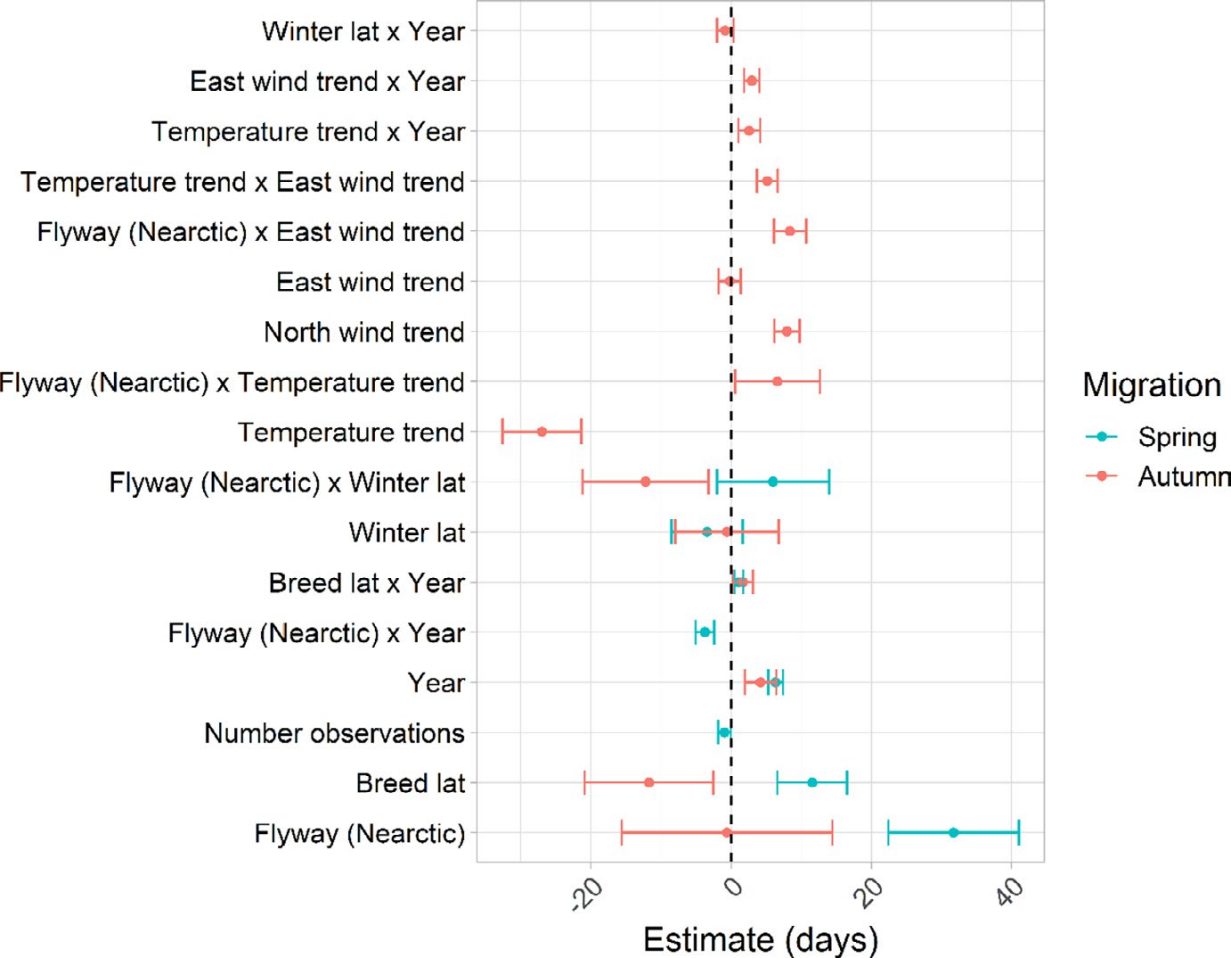
Factors affecting the timing of spring and autumn migration. Positive values of the estimate indicate migration getting later and negative values migration getting earlier. The factors are depicted as the averaged estimates of fixed effects from the models within 2 AICc of the best‐fitting LME. Only variables that were deemed important after model averaging are shown here for clarity; for the full model outputs, see Tables S2 and S3. Horizontal error bars show the standard errors. If a circle and associated error bars do not appear for either spring or autumn migration, this means that the variable was not present in the best‐fitting model list. The intercepts of the models were 75.3 days in spring and 238.8 days in autumn, but were excluded for clarity. Breed lat = breeding latitude index; Winter lat = wintering latitude index

**FIGURE 3:**
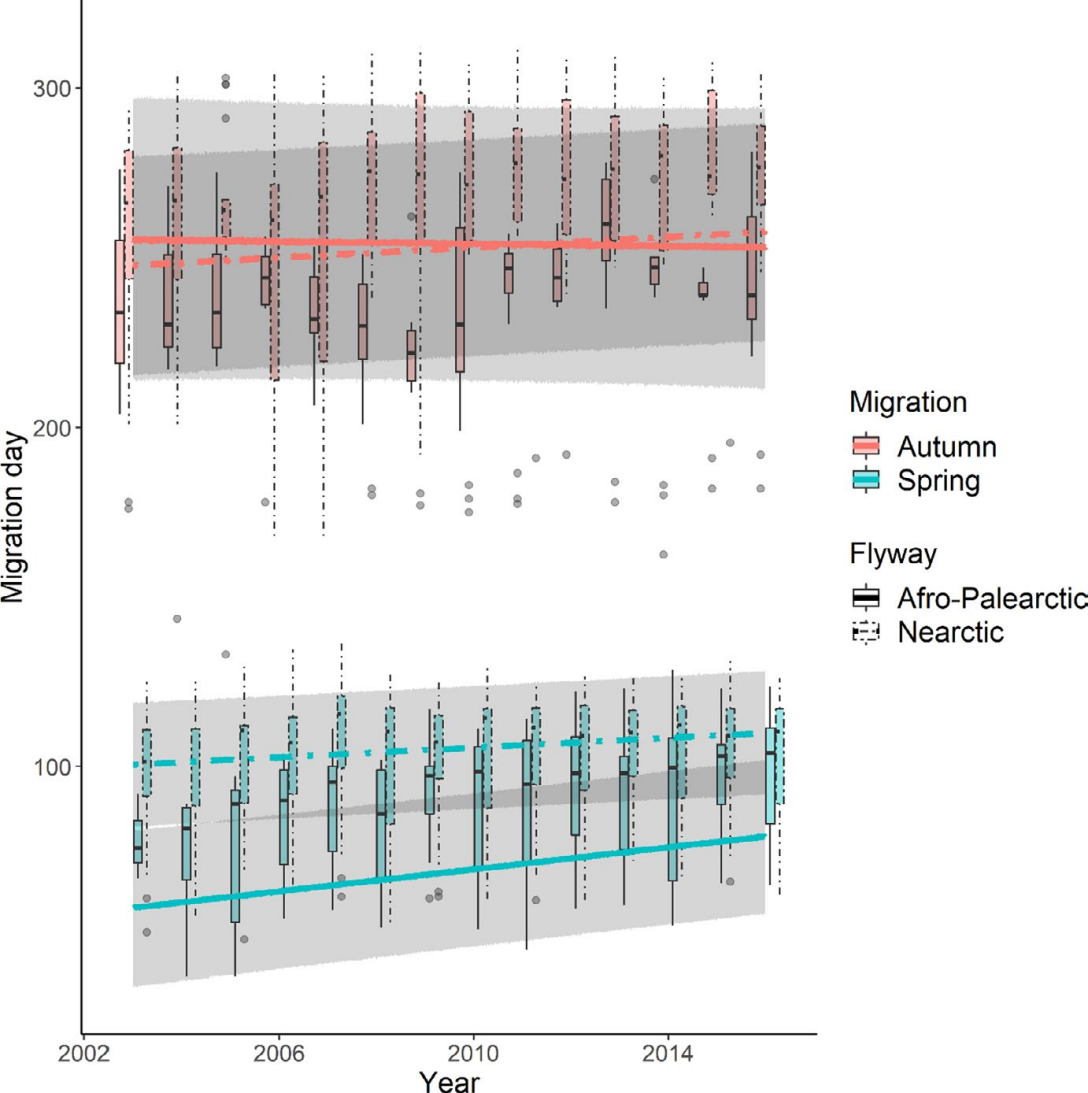
Changes in the timing of spring and autumn migration over time for fifty species of wader, in the Afro‐Palearctic and Nearctic flyways. Boxplots are the distribution of the raw migration day data, which show the median, interquartile range, 1.5 times the interquartile range, and any outliers. Lines show the model averaged predicted relationship from the models within 2 AICc of the best‐fitting LMEs. The ribbons show the 95% prediction intervals of the model averaged fixed effects

We used the “changepoint” package (Killick & Eckley, 2014) available in R (R Core Team, 2019) to implement changepoint detection. We used the Segment Neighbourhood Search algorithm to detect changepoints (Auger & Lawrence, 1989). This allowed us to restrict the number of changes detected, in each year running January through to December, to be two. These correspond to one latitudinal change for spring migration and one latitudinal change for autumn migration, splitting the data into three segments (Figure 1, Figure S1). We obtained two sets of changepoint locations for each year. The first corresponded to changes in mean and the other in mean and variance combined. To obtain these, we used the “cpt.mean” and “cpt.meanvar” functions, respectively. Changes in mean were identified because migration is most logically defined as a latitudinal shift in the mean of a species' distribution over the year. Identifying changes in mean and variance simultaneously was useful because (a) some species have wider wintering ranges than breeding ranges and (b) winter sighting distributions were more variable than breeding ground distributions based on visual inspection of the raw data. We did not identify changes in variance only, because, as explained above, wading bird migration is a shift in mean latitude over the year. We then obtained the day of the year on which the changepoints in latitude occurred for each migration. The dates identified by the two changepoint detection methods were compared with one another in order to refine our estimates of the timing of spring and autumn migrations (Figure 1). We did not use the changepoint estimates if the two methods identified dates that were more than fourteen days apart. After inspecting the raw data, two weeks was considered a suitable threshold to use for the removal of years (number of changepoints removed = 68). We also investigated the influence of this threshold on the mean migration day and found that it had little overall effect (Supplementary material text and Figure 3). In these removed years, the latitudinal data were too variable, particularly in winter, and the analyses could not reliably identify the timing of migration (Supplementary material text and Figure 2). In all other cases, we used the mean of the dates identified by the two methods as the migration date in all subsequent analyses, hereafter referred to as the “migration day.” This made for a better‐defined estimate of changes in the latitudinal data for each year and a more reliable estimate of the timing of spring and autumn migration. The migration days identified were plotted against the raw latitudinal data for all species using time series plots, in order to check that they corresponded to actual shifts in latitude. In all cases, there was close correspondence throughout the year.

Using changepoint analysis to identify the beginning and end of each migration, in some instances, proved problematic. This is because the entire population of a bird species does not migrate simultaneously. This manifests as a slope in the mean latitude of a species' distribution as individuals move at different times between their breeding and wintering regions, and not an abrupt shift (Figure 1). Changes in slope are harder to identify (Baranowski et al., 2019). Detecting changes in mean can be thought of as fitting a step function to the data such that the errors between this step function and the data are minimized. As a result, if there is a slope, and not an abrupt change, the changepoint will often be placed in the center of this slope. As such, the migration days identified using this method approximate the midpoint of migration.

### 2.3 Weather data

#### 2.3.1 Identifying breeding and wintering regions

In order to obtain relevant weather data for each species, we needed to identify their breeding and wintering ranges. For each species, we took the means of all the migration days identified by the changepoint analysis across all years for spring and autumn migration separately. This gave a mean migration day for spring and autumn migration for each species in the study period. The latitudinal distribution of all the sightings reported between these averaged migration days was therefore an index of the breeding distribution; the latitudinal distribution of those reported before and after the average spring and autumn migration days, respectively, was an index of the wintering distribution. However, because the migration days correspond to the midpoint of migration, these sightings spanned part of the migration period also. Therefore, we excluded all the sightings falling outside of the 10th and 90th percentiles of the latitudinal distribution of sightings for each species. The remainder provided indices of the breeding and wintering distribution for each species which were then used to select relevant weather data.

#### 2.3.2 Weather data download

In order to investigate the potential for seasonal change in weather to influence migration day, weather conditions were obtained from ERA‐INterim reanalysis models. ERA‐INterim provides global datasets of past climate variables at an approximately 80‐km resolution and various timescales that are unaffected by changes in method and uses up‐to‐date forecast models (Dee et al., 2011). We obtained weather conditions for the migration days identified by the changepoint analysis in the regions defined as the breeding and wintering areas described above. Data download and processing were carried out in Python V3.8.0 (Sanner, 1999).

For spring migration, we extracted weather data from the wintering area; for autumn, we extracted weather data from the breeding area. This allowed us to investigate if weather variables at the departure location correlated with the timing of migration. Because the migration day identified by the changepoint analysis equates to the middle of migration, individuals will migrate in the weeks before and after the day identified. We therefore retrieved weather data for the entire breeding or wintering region at noon for each day over a forty‐day time window, centered on the migration day identified for each species (see below). Wader species can migrate either diurnally or nocturnally (Lank, 1989), but day‐ and night‐time weather conditions will be highly correlated in our data because our analyses are at large temporal and spatial scales. The weather variables downloaded were northward and eastward wind, and temperature for air pressures of 1,000 hPa, corresponding to sea surface level. The weather variables were averaged over the entire breeding and wintering distribution of each species. We also considered 925, 850, and 750 hPa, corresponding roughly to 760, 1,500, and 2,500 meters above sea level, respectively; all were highly correlated and so only surface‐level data were used. Although birds sometimes migrate at high altitudes (Senner et al., 2018), they are likely to take cues regarding migration from surface‐level weather conditions (Åkesson et al., 2016). We excluded weather conditions over the oceans by applying a land mask. Although migratory birds often cross oceans on migration, they are most likely to take cues from conditions experienced where they are stationary (Åkesson et al., 2016).

#### 2.3.3 Weather trends

For each weather variable, we fitted a linear least‐squares regression over the forty‐day window and used the slope of that line in our models. We chose the forty‐day period because we were investigating changes in migratory behavior across large temporal and spatial scales. Furthermore, most of the individuals of a species are likely to migrate within a window of approximately this length (Horton et al., 2019; Newton, 2010). The rate of change in weather is likely to be crucial for the timing of both spring and autumn migration as individuals take cues from generally improving conditions for migration (i.e., the rate of change in weather conditions), rather than a threshold (Åkesson et al., 2016; Shamoun‐Baranes, Bouten, et al., 2010; Shamoun‐Baranes, Leyrer, et al., 2010). We therefore investigated whether migration was correlated with the change in northward wind, eastward wind, and temperature.

### 2.4 Statistical analyses

We analyzed the factors affecting the timing of spring and autumn migration using linear mixed‐effects models (LMEs). Analyses were carried out in the R environment (R version 3.6.3; R Core Team 2019). Spring and autumn migration days were modeled separately because the influence of life‐history traits and weather are likely to differ between the two (Conklin et al., 2013; McNamara et al., 1998). We only included species for which at least 10 years' worth of data were available in the models, totaling twenty Nearctic species and ten Afro‐Palearctic species in spring, and eighteen Nearctic and six Afro‐Palearctic species in autumn (Table S1).

We fitted the same explanatory variables in the models of changes in the timing of spring and autumn migration. The nonweather variables used were year (fitted as a continuous variable), flyway, the mean breeding and wintering latitude defined using the method described above, and the total number of bird observations reported on the migration day. The latter variable was included to account for the increasing number of observations made over time. The indices of breeding and wintering latitude were included to account for potential differences in the response of species to climate change across latitudinal gradients. The weather conditions included were temperature and northward and eastward wind trends. For each model, we included all two‐way interactions, except for those involving the number of observations. All continuous variables were centered and scaled prior to analyses to allow direct comparisons between fixed effects in the model outputs (Schielzeth, 2010). The weather variables were not detrended because we were specifically interested in understanding changes in their influence over time. Furthermore, the scaling of predictors allows the direct interpretation of variables over and above other model terms (Schielzeth, 2010). However, in order to verify that this did not influence our interpretation of their effects, we also fitted models with detrended weather variables. These models contained the same explanatory variables, but without the two‐way interactions between year and weather trends. They were detrended by using the residuals of separate linear models with the weather trend of interest as the response variable and year as the sole explanatory variable. The effect sizes of the three weather variables and their various interactions in these models were very similar to those obtained without detrending (Tables S2–S5). Species was included as a random effect, and the models were fitted with a Gaussian error distribution. The “lme4” package was used to fit LMEs (Bates et al., 2015). All possible models were fitted and those within 2 AICc of the best‐fitting model were averaged for plotting (Burnham & Anderson, 2002). The full average estimates and adjusted standard errors of the key variables of the best‐fitting models are presented in the results section; the entire table of full model averaged coefficients can be found in the Supplementary material text and Table S8. Models were validated by assessing the normality of residuals and the relationship between the residuals and each explanatory variable.

## 3 RESULTS

**FIGURE 4:**
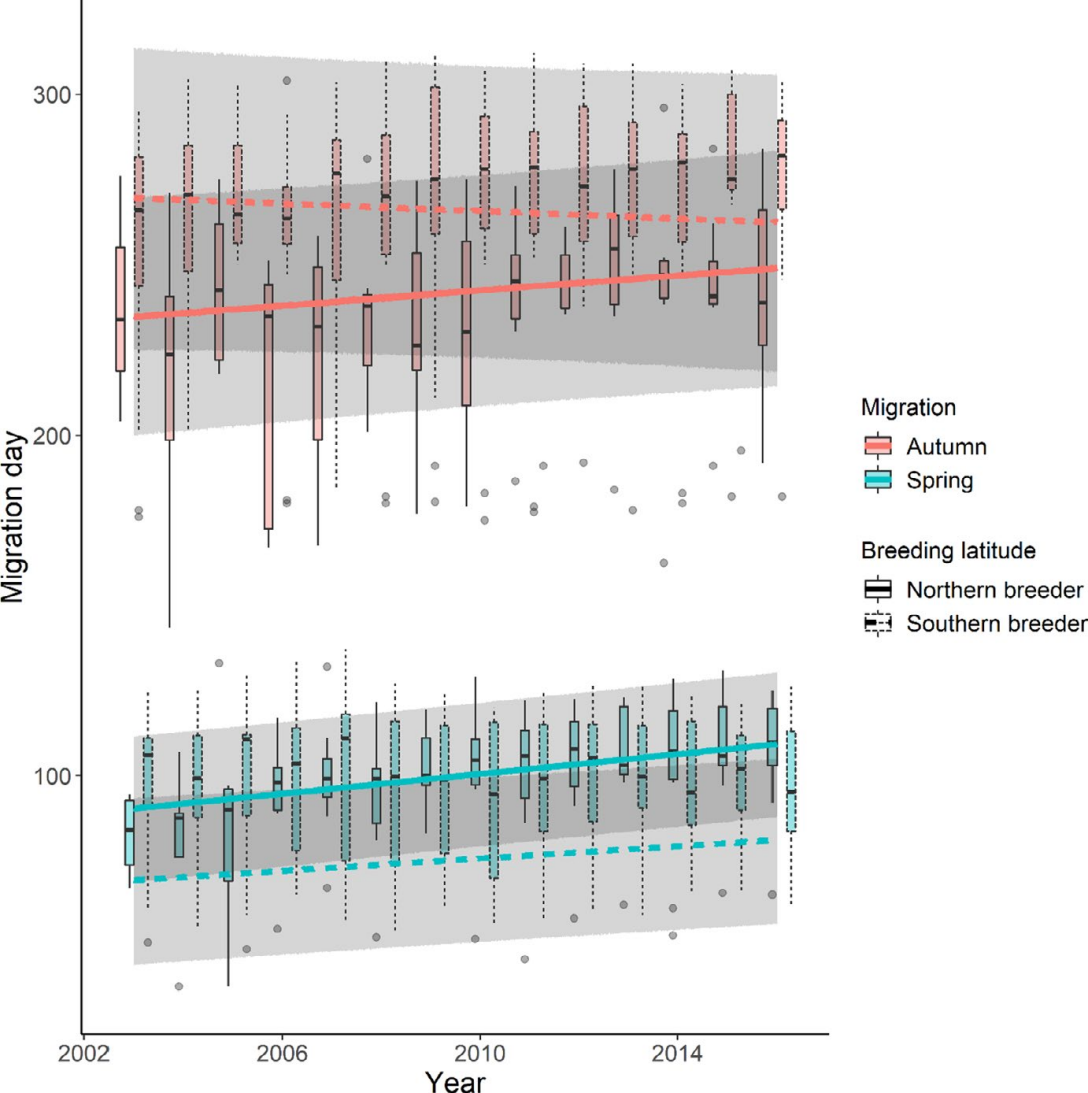
Changes in timing of spring and autumn migration over time for species breeding at northern (58°N) and southern (42°N) latitudes. Boxplots are the distribution of the raw migration day data, which show the median, interquartile range, 1.5 times the interquartile range, and any outliers. Lines show the model averaged predicted relationship from the models within 2 AICc of the best‐fitting LMEs. The ribbons show the 95% prediction intervals of the model averaged fixed effects

The migration day of waders in both spring and autumn became later over the thirteen‐year study period (estimate_spring_ = 6.34 ± 1.06 days, *z*‐value_spring_ = 6.00; Est._autumn_ = 4.21 ± 2.25 days, *z*‐value_autumn_ = 1.84), with changes in spring migration in the Afro‐Palearctic flyway occurring the most rapidly (at approximately 0.5 days/year in the Afro‐Palearctic and 0.2 days/year in the Nearctic; interaction flyway × year, Est._spring × Nearctic_ = −3.73 ± 1.34 days, *z*‐value = 2.78; Figures 2 and 3, Tables S2 and S3). In autumn, there was no influence of flyway over time (interaction flyway × year (Est._autumn × Nearctic_ = 0.54 ± 1.90 days, *z*‐value = 0.29; Figures 2 and 3, Tables S2 and S3), with migration becoming later by approximately 0.3 days/year (Figures 2 and 3). Spring migration was about thirty days earlier in the Afro‐Palearctic than the Nearctic flyway (Est._Nearctic_ = 31.73 ± 9.34 days, *z*‐value = 3.40). Breeding latitude was an important predictor in the models of both migrations. Northern breeders migrated later in spring and slightly earlier in autumn than those breeding at more southerly latitudes, consistent with shorter breeding seasons at northerly latitudes (Est._spring_ = 11.55 ± 4.97 days, *z*‐value = 2.32; Est._autumn_ = −11.70 ± 9.21 days, *z*‐value_autumn_ = 1.27; Figures 2 and 4). The timing of spring migration for both northern and southern breeders became slightly later over time, but changes occurred more rapidly in the former (at approximately 1.47 days/year for northern breeders and 0.92 days/year for southern breeders; interaction breeding latitude × year, Est._spring_ = 1.08 ± 0.65 days, *z*‐value_spring_ = 1.65); in autumn there was no difference in the change in the timing of migration between northern and southern breeders (interaction breeding latitude × year, Est._autumn_ = 1.67 ± 1.46 days, *z*‐value_autumn_ = 1.15; Figure 4).

### 3.1 Effects of weather

**FIGURE 5:**
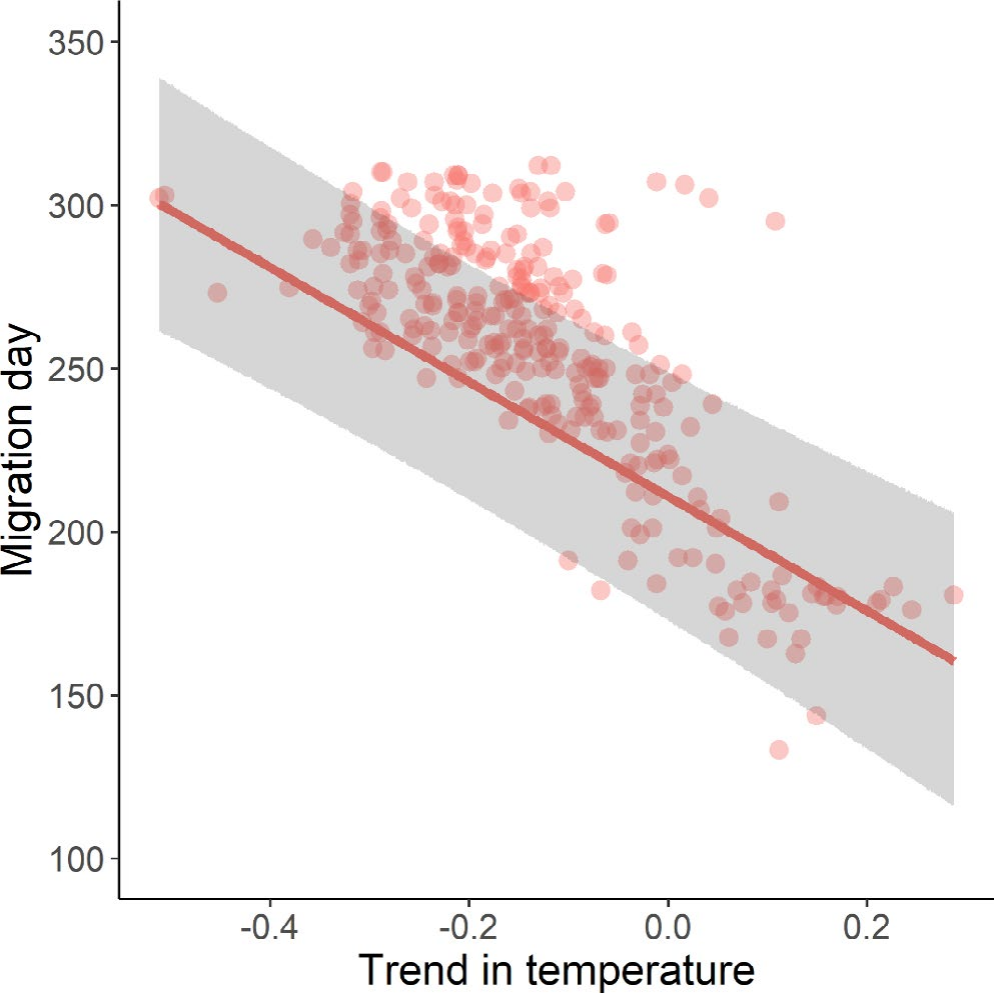
Relationship between the timing of autumn migration and changes in temperature. The *x*‐axis is the slope from the linear least‐squares regression. Closed circles show the raw data; the line shows the model averaged predicted relationship from the models within 2 AICc of the best‐fitting LME. The ribbon shows the 95% prediction intervals of the model averaged fixed effects. Trend in temperature data are the slopes of linear least‐squares regressions of temperature against day of the year for the twenty‐day window surrounding a migration day

**FIGURE 6:**
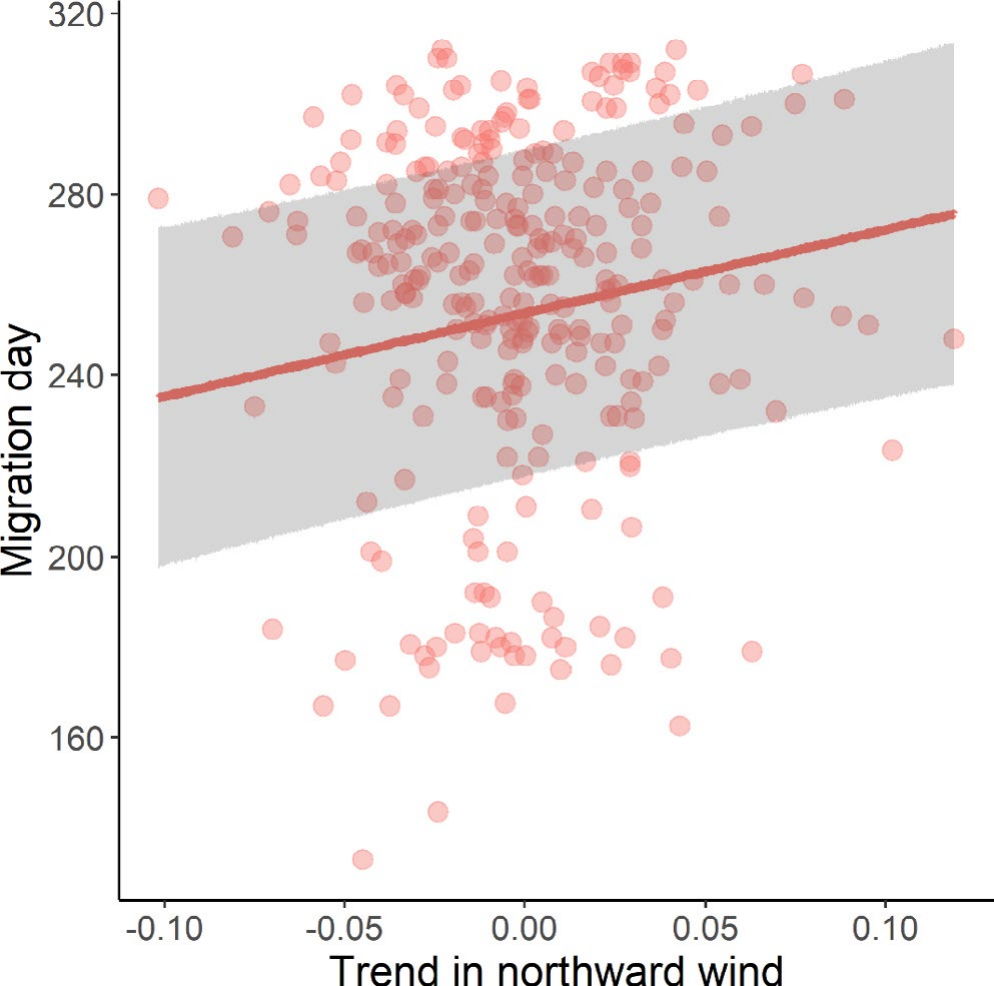
Relationship between the timing of autumn migration and changes in northward winds. The *x*‐axis is the slope from the linear least‐squares regression. Circles show the raw data; the line is the model averaged predicted relationship from the models within 2 AICc of the best‐fitting LME. The ribbon shows the 95% prediction interval of the model averaged fixed effects. Trend in northward wind data are the slopes of linear least‐squares regressions of temperature against day of the year for the twenty‐day window surrounding a migration day

Weather variables were important correlates of autumn migration days only; in spring, there were no correlations between migration days and weather (Figure 2). Autumn migration was earlier when temperatures became warmer more quickly (Est. = −26.97 ± 5.63 days, *z*‐value = 4.79; Figure 5). Autumn migration also occurred later when headwinds were increasing, and earlier when tailwinds were increasing (Est. = 7.97 ± 1.80 days, *z*‐value = 4.42; Figure 6). Although weak, the effect of eastward wind differed between the flyways; stronger eastward winds were correlated with later migration days in the Nearctic but not the Afro‐Palearctic flyway (interaction flyway × eastward wind, Est. = 8.40 ± 2.32 days, *z*‐value = 3.63; Figure 2). The effects of both the temperature and eastward wind trends changed in the same way over time; migration became later over time more quickly when temperatures became warmer more quickly (interaction temperature × year, Est. = 2.58 ± 1.55 days, *z*‐value = 1.67) and when eastward wind became stronger more quickly (interaction eastward wind trends × year, Est. = 2.95 ± 1.10 days, *z*‐value = 2.67) than when these were getting colder and weaker, respectively (Figure 2).

Several potential biases in the sightings data could have influenced the results of our models, such as changes in the timing of sightings within each migration period or changes in the proportion and the rate of increase of sightings reported from different latitudes over the study period. We investigated these but found that they were unlikely to have driven the results of our models (Supplementary Material Text and Figures).

## 4 DISCUSSION

We found that both spring and autumn migration in waders are likely to have become later over time in the Nearctic and Afro‐Palearctic flyways, which is in contrast with the results from many studies of population‐level phenology in migratory species (Gunnarsson & Tómasson, 2011; Lehikoinen et al., 2004). However, patterns in the timing of migration are likely to vary at different spatial scales (Chambers et al., 2014; Kelly & Horton, 2016). Furthermore, the responses to climate change, and the mechanisms driving these responses, could show significant intraspecific variation. Indeed, our results still allow for the timing of migration to become earlier in a subset of individuals but show that overall timings may be shifting later, perhaps due to changes in species' ranges.

Range increases could cause flyway‐level migration to become later in two ways: (a) individuals migrating further distances due to the colonization of new habitats (Howard et al., 2018), and (b) individuals breeding further north migrating later than those breeding at more southerly locations, as we found (see below). Cross‐species meta‐analyses have revealed northward shifts of bird species' ranges at up to 16.9 km per decade (Chen et al., 2011; Parmesan & Yohe, 2003). Furthermore, contractions at the warm limit of species' ranges occur at a slower rate than expansions at the cold limit (Parmesan et al., 1999; Virkkala & Lehikoinen, 2014), thereby increasing global range size. The flyway‐level timing of species' migrations could even become later without overall range shifts, if the proportion of individuals migrating to higher latitudes increases. This would manifest as the migration day becoming later, just as we found in our analysis, because individuals take longer to reach their breeding sites.

We found that the flyway‐level spring migration of northerly breeders became later over time than that of southerly breeders, which supports the idea that range shifts might be driving the timing of migration becoming later. Range shifts occur more rapidly for northerly breeding species than southerly species because of greater temperature increases at high latitudes, resulting in a higher proportion of individuals traveling to northerly latitudes (Chen et al., 2011; Tingley & Huybers, 2013). For species with high adult site fidelity, it is possible that these expansions may be driven by juveniles colonizing new areas (Gill et al., 2014, 2019), although site fidelity is likely to vary substantially between species. Additionally, climate change has caused warming and increased climate variability in recent decades, particularly between 20° and 50°N (Cohen et al., 2014). Variability in weather could increase the strength of selection on individuals such that only individuals in the best body condition are able to arrive early. For example, while the fittest (earliest) migrants may advance their migration, relatively more poor‐quality individuals may be affected by weather events, thereby causing overall timings to become later (Duijns et al., 2017; Shamoun‐Baranes, Bouten, et al., 2010; Shamoun‐Baranes, Leyrer, et al., 2010). Furthermore, birds breeding at high latitudes are exposed to weather conditions for a longer period of time during migration, meaning that they may be more susceptible to weather effects than southerly breeders.

Our results appear to contradict those of several studies carried out at the population level (Gunnarsson & Tómasson, 2011; Lehikoinen et al., 2004). The mechanisms driving changes at population and flyway‐level scales are likely to differ given that the effects of climate change vary globally (Chmura et al., 2019). This could influence the results from population‐level studies, as even those combining data from multiple populations do not account for changes occurring to areas outside of study regions. It is also possible that our results are not directly comparable with many studies carried out at the individual level, as our changepoints analysis identified the midpoint of the migration window. Even with the start or end of the migratory window becoming earlier at an individual level, the central point could still shift later. To our knowledge, the only other study to investigate changes in the timing of migration at a flyway scale found contrasting results to ours (Horton et al., 2019). However, our analysis is restricted to waders and uses sightings of each species rather than radar data of all species combined. Furthermore, our dataset corresponds to only the latter half of theirs, during which they found a decrease in the trend of earlier spring migration, and that autumn migration was becoming later.

### 4.1 The influence of weather on migratory timing

Weather conditions in spring are thought to be less important for migratory birds than those in autumn due to the time constraints associated with breeding (McNamara et al., 1998). Individuals in spring are likely to continue migration regardless of large‐scale weather trends, instead being affected by short, extreme events, which our trend variables would not identify (Conklin et al., 2013; Loonstra et al., 2019; McNamara et al., 1998). Conversely, autumn migration is likely to be more strongly influenced by trends in weather conditions and the speed at which chicks fledge (Conklin et al., 2013; Shamoun‐Baranes, Bouten, et al., 2010; Shamoun‐Baranes, Leyrer, et al., 2010). Indeed, we found that warming temperatures over a forty‐day window were strongly correlated with earlier autumn migrations but found no such patterns in spring. Warmer temperatures during breeding increase insect abundance and will improve conditions for chicks (Townsend et al., 2013). Wading bird species have precocial offspring and increased insect abundance will benefit foraging success (McGowan et al., 2002), likely resulting in faster fledging. The autumn migration of birds became later at a faster rate when temperature trends were more positive, which may be due to high temperatures lasting later into the year allowing more time for replacement clutches (Morrison et al., 2019).

Finally, we found that increases in headwinds were negatively correlated with autumn migration. Studies have shown that individuals avoid headwinds during migration and wait for improved flight conditions to maximize flight efficiency (Åkesson & Hedenström, 2000). Crosswinds were only important in the Nearctic flyway, perhaps because strong eastward winds would push individuals in Central America out into the Gulf of Mexico, which could be fatal. The effects of wind conditions on migratory birds, and how these are likely to change, are complex. While autumn headwinds are projected to increase (La Sorte et al., 2019), crosswinds may decrease (La Sorte & Fink, 2017). These changes are likely to have important consequences which could differ between individuals and species depending on their size and migratory behavior (Anderson et al., 2019).

Understanding how individual‐level mechanisms drive flyway‐level responses to climate change is important for migratory bird conservation and for investigating changes in ecosystem services and functioning (Wilcove & Wikelski, 2008). More work incorporating citizen science, weather surveillance radar data, and detailed information from individual populations spread across entire geographic ranges should be particularly insightful (Kelly & Horton, 2016; Wilcove & Wikelski, 2008).

## CONFLICT OF INTEREST

The authors have no conflict of interest to declare.

## AUTHOR CONTRIBUTIONS

**Thomas O. Mondain‐Monval:** Conceptualization (equal); data curation (equal); formal analysis (lead); investigation (lead); methodology (equal); validation (equal); visualization (lead); writing‐original draft (lead); writing‐review & editing (equal). **Matt Amos:** Data curation (equal); formal analysis (supporting); resources (equal); writing‐review & editing (equal). **Jamie‐Leigh Chapman:** Formal analysis (supporting); investigation (supporting); methodology (equal); resources (equal); validation (supporting); writing‐review & editing (equal). **Andrew MacColl:** Conceptualization (equal); funding acquisition (equal); investigation (supporting); project administration (supporting); supervision (equal); writing‐review & editing (equal). **Stuart P. Sharp:** Conceptualization (equal); formal analysis (equal); funding acquisition (equal); investigation (equal); methodology (equal); project administration (lead); supervision (lead); validation (equal); writing‐original draft (equal); writing‐review & editing (equal).

## Supporting information

Supplementary Material

## Data Availability

All data are freely available from the eBird Citizen Science Database, https://ebird.org/science/use‐ebird‐data/download‐ebird‐data‐products.
