# Supplementary material for: Flyway‐scale analysis reveals that the timing of migration in wading birds is becoming later
Source: Ecol Evol. 2021 Sep 28;11(20):14135–45. doi: 10.1002/ece3.8130 (PMC8525091; doi:10.1002/ece3.8130)
Supplement: Supplementary file 1 — Supplementary Material [file ECE3-11-14135-s001.zip › Supplementary material/Supplementary Material Text and Figures.docx]

# Supplementary Material

We chose to omit migrations in which the changepoints removed by the two methods (mean and mean variance) were over two weeks apart. To ensure that this cut off did not bias our results, we compared the timing of the changepoints that were kept (<15 days apart) to those that were omitted (>14 days apart). The distributions of the changepoints that were kept and removed were broadly similar for both changepoints methods, with only removed changepoints identified by the mean variance method in autumn being slightly earlier than those which were kept (Supplementary material figure 2). This is consistent with the fact that species with few data had wide latitudinal distributions, particularly in winter, as mentioned in the main text. To investigate further our choice of the two-week cut off value, we compared the mean migration day when using different values. For each species and migration period, we calculated the mean migration day across the two different changepoints methods when omitting data that fell different distances apart; dates were omitted when they were more than {0, 4, 9, 14, 19, 24, 29, 34, 39} days apart. There were no consistent differences in the mean migration days when omitting data based on these different cut off values (Supplementary material figure 3).

## Changes in the number, timing and distribution of observations

Several factors could influence the model results. One of the major issues with the eBird dataset is that the amount of data collected has increased dramatically over time. However, in our models the number of observations made in any given year was negatively correlated with the migration day in spring and no correlation was found in autumn (Figure 2). It is therefore unlikely that the migration day becoming later over time was driven by changes in the number of observers.

The timing of sightings within the migration periods could influence the identification of the migration day by changepoint analysis. If sightings were reported during later stages of migration over the study period, which could happen due to the increase in observers, then the migration day might become later. To test this, we filtered the raw sightings data to include only those reported within each migration period: between March 1^st^ and June 28^th^ for spring and between June 28^th^ and September 7^th^ for autumn. These dates were chosen after visual inspection of the raw latitudinal data. Then, we fitted an LME with the day of sighting (as a Julian date) as the response variable, year as a continuous fixed effect (scaled and centred) and species as a random effect. The timing of sightings became earlier over those periods for both migrations, not later (spring model: estimate = -1.05, se = 0.01, t-value = -78.34, *n* = 4 306 007; autumn model: estimate = -1.80, se = 0.01, t-value = -156.5, *n* = 4 409 989).

Changes in the latitudinal distribution of observers over time could also have caused the migration days to become later. In spring, proportionally more sightings could have been reported at higher latitudes in later years because ornithologists started visiting locations further north. In autumn, proportionally more sightings from lower latitudes in later years would have the same effect. In order to test this, for each migration period annually, we split all the sightings data into ten-degree latitudinal bands covering the entire range of all species in the dataset (55°S to 85°N). We then plotted the number of sightings reported in each latitudinal band for each month and year separately. The proportion of sightings in each band did not vary substantially throughout the study period in different months. Importantly, the proportion of sightings reported from high and low latitudes did not increase over the study period in any of the months investigated (Supplementary material Figure 3).

We then fitted two LMEs, one for spring and one for autumn, to investigate the rates of change in the number of sightings from different latitudes from early and late stages of the season. We determined the number of sightings reported on each day and fitted that as the response variable with species as a random intercept. As explanatory variables, we fitted the three-way interaction between day of the year (as a Julian date), latitudinal band and year, and a second, similar, three-way interaction with year fitted as a quadratic term because the number of sightings were likely to have increased non-linearly over the study period. The three-way interaction was important in the spring model but less so in the autumn model (Supplementary material Tables 6 and 7). In spring, the number of sightings at late stages of the season from low latitudes increased at a faster rate over time than from high latitudes; the opposite was true at early stages of the season (Supplementary material Figure 4). This suggests that changes in the distribution of sightings over the study period were unlikely to have resulted in the timing of spring migration becoming later in our changepoints analyses. In autumn, increases in the number of sightings happened at similar rates at both high and low latitudes but showed opposite patterns to those in spring. The number of sightings increased more rapidly at late than at early stages of the season (Supplementary material Figure 4).

## Supplementary Material Figures


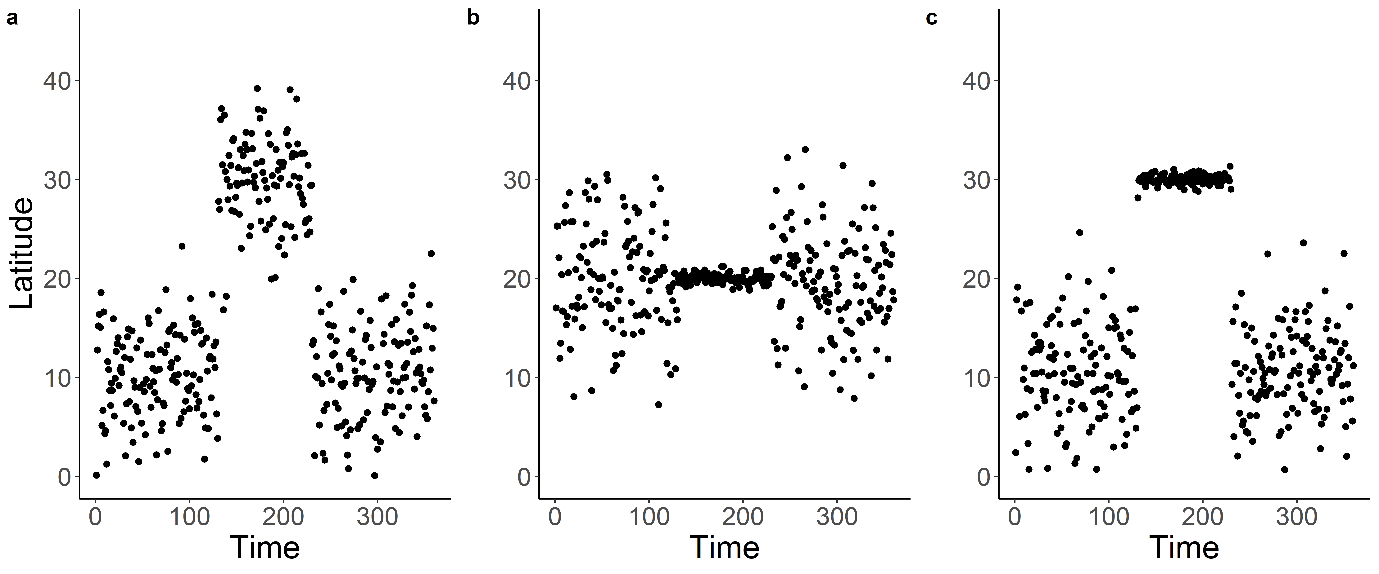


**Supplementary Material Figure 1** Example data with changes in (a) mean, (b) variance and (c) mean and variance combined. Each dataset has two changepoints, splitting the data into three segments.


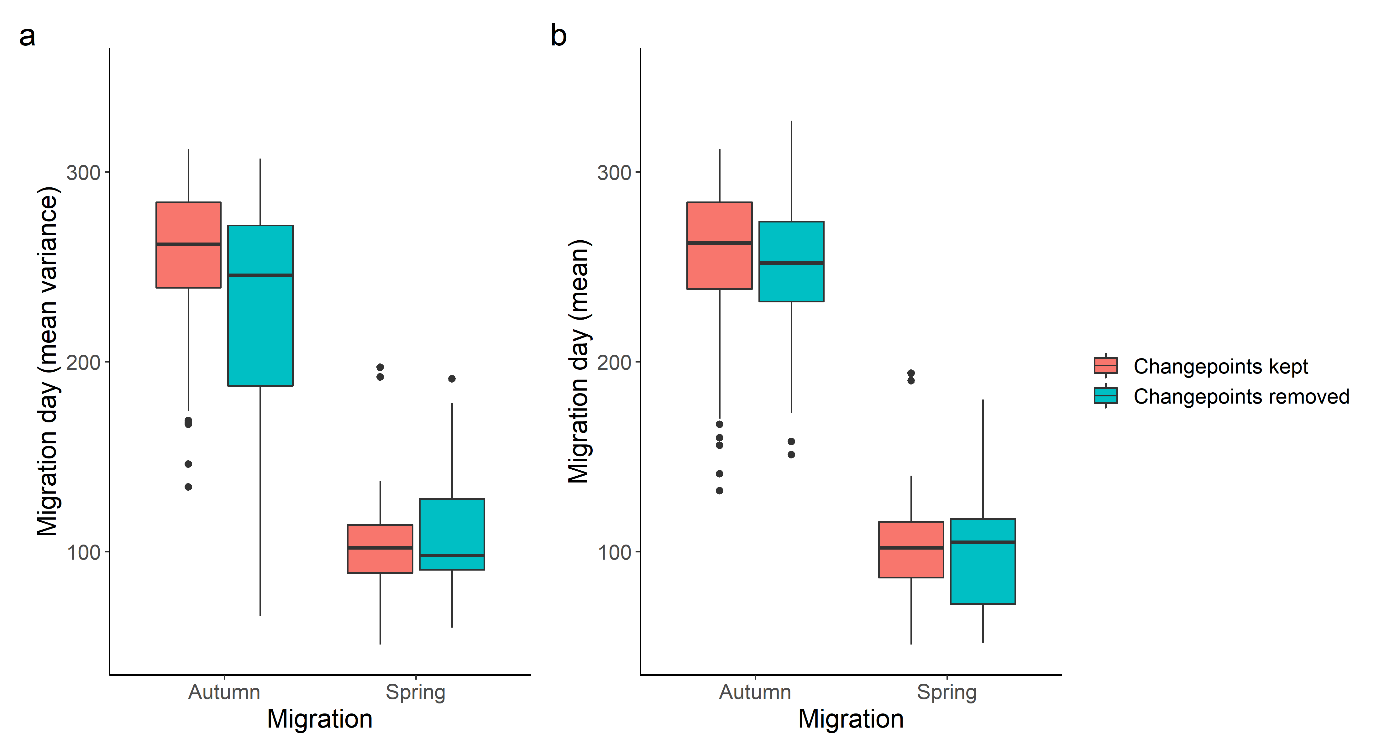


**Supplementary Material Figure 2** Distributions of the changepoints which were removed by the two-week cut off for (a) the mean variance and (b) the mean changepoints identification methods.


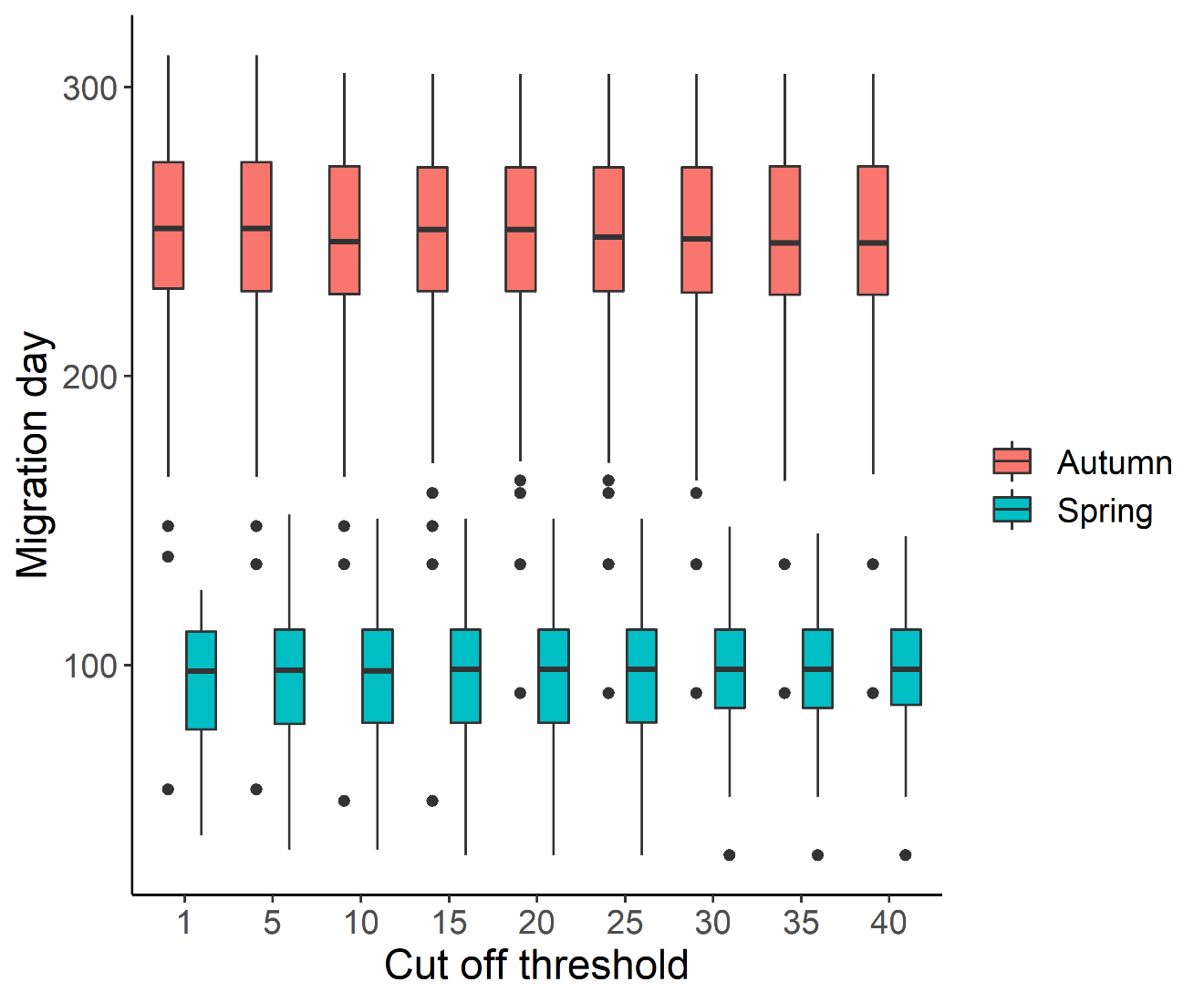


**Supplementary material figure 3** Comparing the effects of changes in the number of cut off days on the mean migration day in spring and autumn.


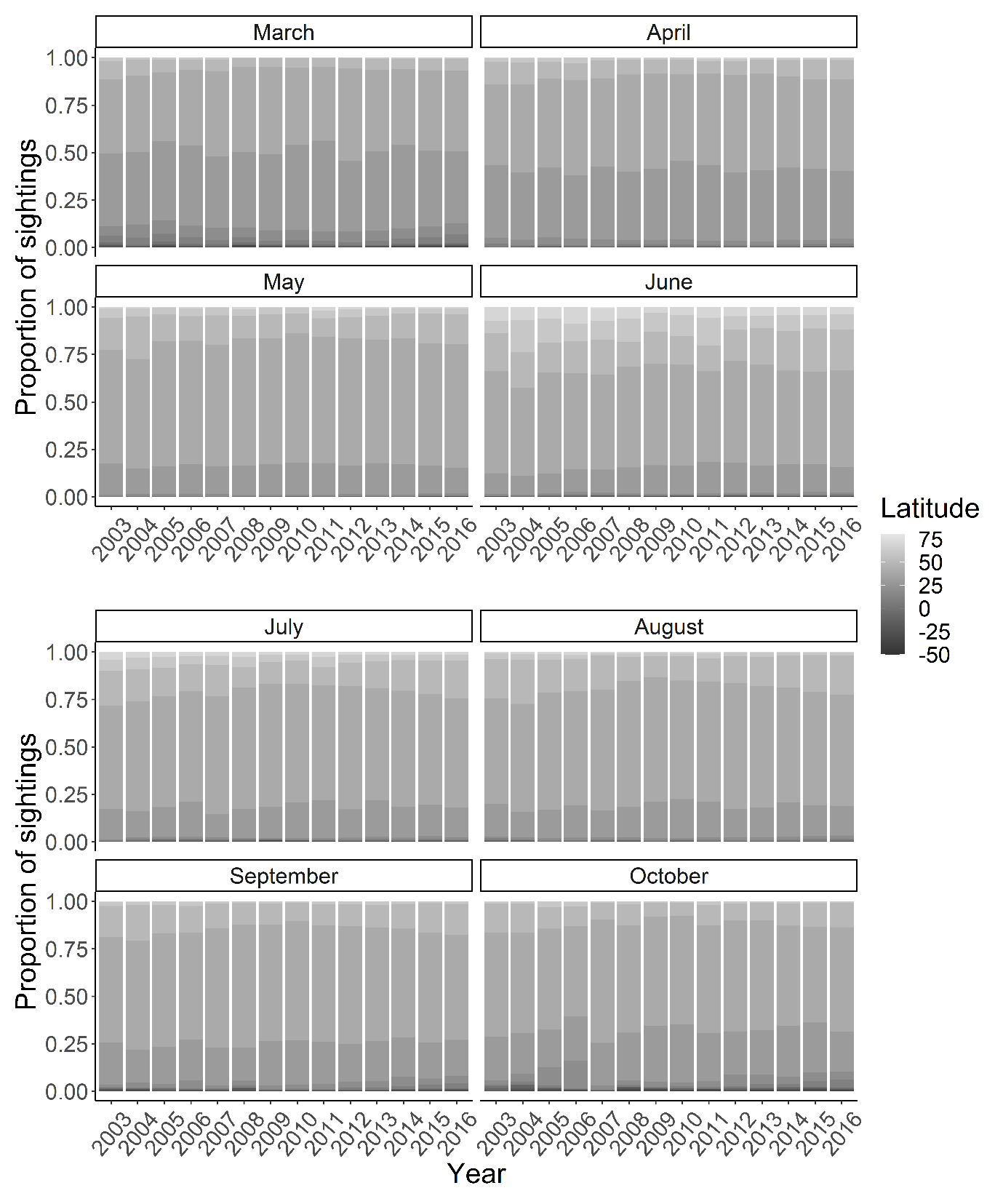


**Supplementary Material Figure 3** The proportions of sightings reported at different latitudes over the study period during the spring (top panels, March – June) and autumn (bottom panels, July – October) migration periods.


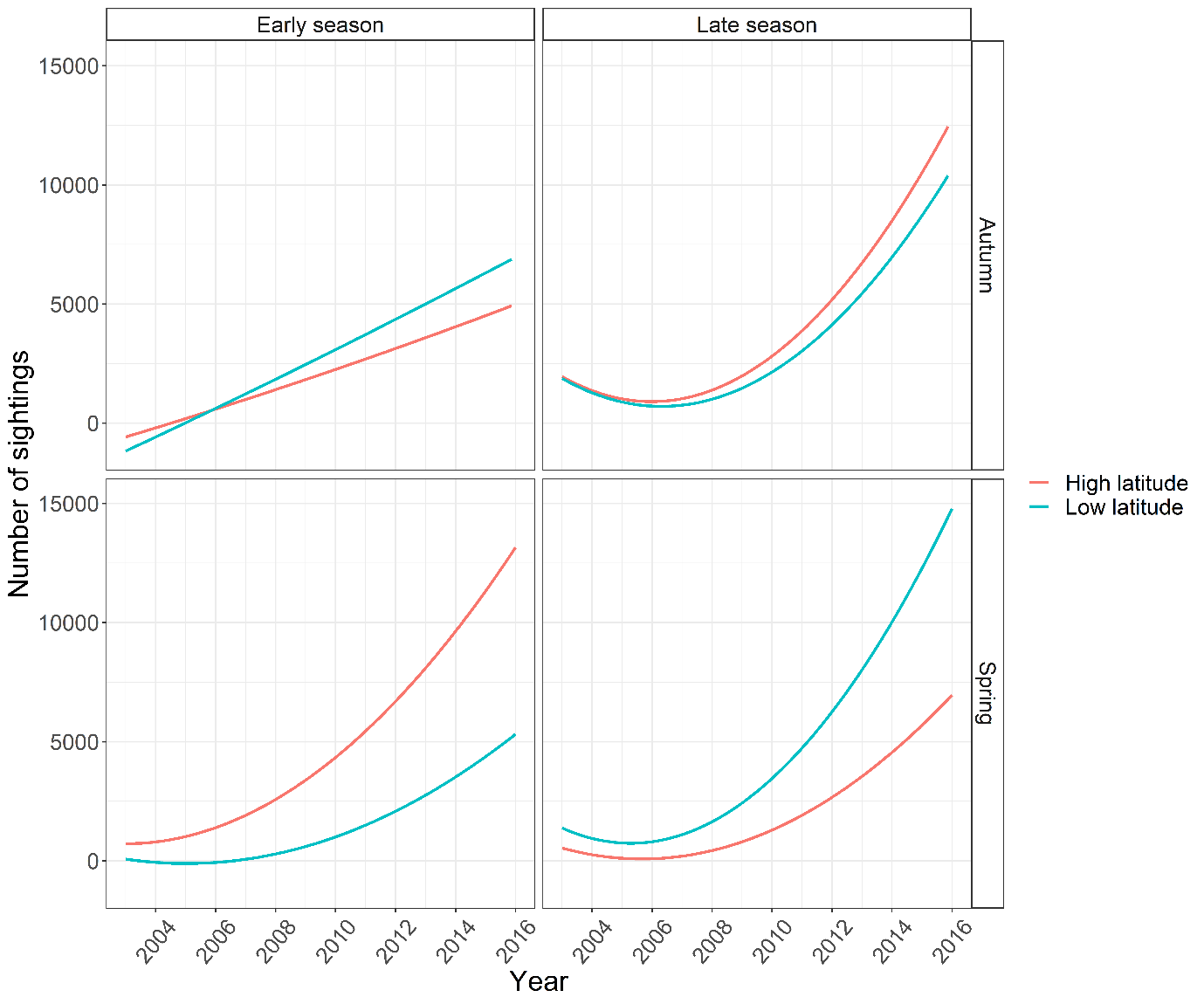


**Supplementary Material Figure 4** Differences in the number of sightings reported to eBird in spring and autumn as a factor of stage of the season (fitted as day of the year as a Julian date), latitude and year. Lines are predictions from linear mixed effects models. High (48˚N) and low (26˚N) latitude, and early (spring = 2^nd^ April, autumn = 25^th^ July) and late (spring = 29^th^ May, autumn = 12^th^ September) stages of the season, are two standard deviations above and below the mean latitude (37˚N) and mean stage of the season (spring = 30^th^ April, autumn = 19^th^ August).

| Supplementary material Table 8 Model averaged coefficients of the models within 2AICc of the best-fitting model for spring and autumn migration. | | | | | |
| --- | --- | --- | --- | --- | --- |
| Fixed effects | Estimate | Adjusted SE | z-value | Importance | Migration |
| (Intercept) | 75.29 | 7.61 | 9.89 | NA | spring |
| Flyway (Nearctic) | 31.73 | 9.34 | 3.40 | 1.00 | spring |
| Breed lat | 11.55 | 4.97 | 2.32 | 1.00 | spring |
| Number observations | -0.92 | 0.91 | 1.01 | 1.00 | spring |
| Year | 6.34 | 1.06 | 6.00 | 1.00 | spring |
| Flyway (Nearctic) x Year | -3.73 | 1.34 | 2.78 | 0.92 | spring |
| Breed lat x Year | 1.08 | 0.65 | 1.65 | 0.69 | spring |
| Winter lat | -3.43 | 5.08 | 0.68 | 0.44 | spring |
| Flyway (Nearctic) x Winter lat | 5.99 | 8.02 | 0.75 | 0.44 | spring |
| Flyway (Nearctic) x Breed lat | -1.36 | 4.51 | 0.30 | 0.23 | spring |
| Temperature trend | 0.82 | 2.03 | 0.40 | 0.17 | spring |
| Flyway (Nearctic) x Temperature trend | -0.78 | 2.09 | 0.37 | 0.16 | spring |
| North wind trend | 0.31 | 0.87 | 0.36 | 0.15 | spring |
| Flyway (Nearctic) x North wind trend | -0.33 | 0.94 | 0.36 | 0.15 | spring |
| East wind trend | -0.05 | 0.22 | 0.25 | 0.14 | spring |
| (Intercept) | 238.76 | 14.35 | 16.64 | NA | autumn |
| Flyway (Nearctic) | -0.59 | 15.05 | 0.04 | 1.00 | autumn |
| Breed lat | -11.70 | 9.21 | 1.27 | 1.00 | autumn |
| Temperature trend | -26.97 | 5.63 | 4.79 | 1.00 | autumn |
| East wind trend | -0.20 | 1.57 | 0.13 | 1.00 | autumn |
| North wind trend | 7.97 | 1.80 | 4.42 | 1.00 | autumn |
| Winter lat | -0.58 | 7.39 | 0.08 | 1.00 | autumn |
| Year | 4.21 | 2.25 | 1.87 | 1.00 | autumn |
| Flyway (Nearctic) x Temperature trend | 6.63 | 6.05 | 1.10 | 1.00 | autumn |
| Flyway (Nearctic) x East wind trend | 8.40 | 2.31 | 3.63 | 1.00 | autumn |
| Flyway (Nearctic) x Winter lat | -12.20 | 9.01 | 1.35 | 1.00 | autumn |
| Breed lat x Year | 1.67 | 1.46 | 1.15 | 0.92 | autumn |
| Temperature trend x East wind trend | 5.18 | 1.47 | 3.52 | 0.84 | autumn |
| Temperature trend x Year | 2.58 | 1.55 | 1.67 | 0.74 | autumn |
| East wind trend x Year | 2.95 | 1.10 | 2.67 | 0.72 | autumn |
| Winter lat x Year | -0.84 | 1.18 | 0.72 | 0.46 | autumn |
| Flyway (Nearctic) x Breed lat | -5.83 | 10.10 | 0.58 | 0.37 | autumn |
| Temperature trend x North wind trend | 0.66 | 1.24 | 0.53 | 0.33 | autumn |
| Flyway (Nearctic) x North wind trend | -0.65 | 1.75 | 0.37 | 0.24 | autumn |
| Flyway (Nearctic) x Year | 0.54 | 1.90 | 0.29 | 0.20 | autumn |
| North wind trend x Year | -0.28 | 0.77 | 0.37 | 0.19 | autumn |
| Number observations | 0.51 | 1.18 | 0.43 | 0.12 | autumn |
| Breed lat x Winter lat | -0.35 | 1.90 | 0.18 | 0.07 | autumn |
| East wind trend x North wind trend | -0.03 | 0.24 | 0.11 | 0.03 | autumn |
